# Meal and habitual dietary networks identified through Semiparametric Gaussian Copula Graphical Models in a German adult population

**DOI:** 10.1371/journal.pone.0202936

**Published:** 2018-08-24

**Authors:** Carolina Schwedhelm, Sven Knüppel, Lukas Schwingshackl, Heiner Boeing, Khalid Iqbal

**Affiliations:** 1 Department of Epidemiology, German Institute of Human Nutrition Potsdam-Rehbruecke (DIfE), Nuthetal, Germany; 2 NutriAct – Competence Cluster Nutrition Research Berlin-Potsdam, Nuthetal, Germany; Univerity of Salzburg, AUSTRIA

## Abstract

Gaussian graphical models (GGMs) are exploratory methods that can be applied to construct networks of food intake. Such networks were constructed for meal-structured data, elucidating how foods are consumed in relation to each other at meal level. Meal-specific networks were compared with habitual dietary networks using data from an EPIC-Potsdam sub-cohort study. Three 24-hour dietary recalls were collected cross-sectionally from 815 adults in 2010–2012. Food intake was averaged to obtain the habitual intake. GGMs were applied to four main meals and habitual intakes of 39 food groups to generate meal-specific and habitual dietary networks, respectively. Communities and centrality were detected in the dietary networks to facilitate interpretation. The breakfast network revealed five communities of food groups with other vegetables, sauces, bread, margarine, and sugar & confectionery as central food groups. The lunch and afternoon snacks networks showed higher variability in food consumption and six communities were detected in each of these meal networks. Among the central food groups detected in both of these meal networks were potatoes, red meat, other vegetables, and bread. Two dinner networks were identified with five communities and other vegetables as a central food group. Partial correlations at meals were stronger than on the habitual level. The meal-specific dietary networks were only partly reflected in the habitual dietary network with a decreasing percentage: 64.3% for dinner, 50.0% for breakfast, 36.2% for lunch, and 33.3% for afternoon snack. The method of GGM yielded dietary networks that describe combinations of foods at the respective meals. Analysing food consumption on the habitual level did not exactly reflect meal level intake. Therefore, interpretation of habitual networks should be done carefully. Meal networks can help understand dietary habits, however, GGMs warrant validation in other populations.

## Introduction

Diet-disease studies frequently evaluate dietary patterns using data reduction techniques (such as and principal component, PCA, or cluster analysis) based on habitual intake. From habitual intake, defined as long-term average, we cannot imply which foods are eaten together. Therefore, our understanding of how dietary patterns arise from food intake is limited. The composition of meals is influenced by personal beliefs and preferences, by social, cultural, geographical, and economic factors, among others [1,2]. Such influences may affect meal intakes, which in turn may affect habitual dietary patterns. Therefore, considering population-specific meal differences, a healthy and an unhealthy dietary pattern might not be formed in the same way in different populations. Analysing food consumption and relationships between foods on the meal level can help to better understand how foods are consumed in relation to each other. This knowledge can be useful for shaping understandable meal-based dietary advice easily adaptable by the public.

Exploratory methods can also be applied to meal-specific data. For instance, *Woolhead et al*. identified 12 meal types from PCA [3]. However, PCA-derived dietary patterns are difficult to interpret as the interrelation between foods is not fully elucidated [4]. Probabilistic Graphical methods such as networks derived through Gaussian Graphical Models (GGMs) offer an insight into the relation between the dietary components and can help understand how foods are consumed in relation to each other during meals. These methods construct conditional independence networks between highly correlated variables in a dataset [5]. GGMs are commonly used in research areas such as omics [6,7] and psychopathology [8,9]. In the field of nutritional epidemiology, these methods have been previously used to construct and visualize dietary networks in specific populations [10]. Semiparametric Gaussian Copula Graphical Models (SGCGMs), a nonparametric extension to GGMs, can be used to analyse skewed data, as is often the case with dietary data [11]. These methods applied to dietary data may help to identify conditional intakes of different foods at meal-level and how those foods appear in habitual dietary patterns.

In this study, we estimated and described meal and habitual dietary networks derived through SGCGMs in a study sample of German adults and compare the relations found in meal networks to the ones present in the habitual network. This study will help to better understand the interrelation of foods consumed at meals and provide an insight about information lost or retained when we perform similar analyses using averaged (habitual) daily dietary data.

## Methods

### Sample size

Data collected between 2010 and 2012 from a validation sub-study within the European Prospective Investigation into Cancer and Nutrition (EPIC)-Potsdam cohort were used for this study; 815 men and women participated in this sub-study. After exclusion of one participant due to dementia, a total of 814 participants were included in our analyses (S1 Fig). More details about this study design are available elsewhere [12]. The Ethics Committee of the Medical Association of the State of Brandenburg provided ethical approval. All participants gave their written informed consent.

### Dietary assessment

Within a year, participants provided up to three 24-hour dietary recalls (24hDR) (5 participants had two 24hDRs and 3 participants had only one 24hDR) using EPIC-Soft [13]. A total of 2,431 24hDRs were collected. During the first visit in the study centre, the first 24hDR was recorded. The following 24hDRs were collected via telephone on randomly chosen days. All recalls were performed by trained interviewers. Food intake data were recorded in 11 eating occasions throughout one day (S1 Table).

### Assessment of other variables

Body weight and height were measured in the study centre during the participants’ first visit. Body mass index (BMI) was calculated as the ratio of weight in kg to height squared in meters. Study participants wore a combined heart rate and uniaxial movement sensor (Actiheart, CamNtech, Cambridge, UK) continuously for one week. Physical activity was then calculated as the total energy expenditure to resting energy expenditure ratio [14].

### Modelling food intake

Food intake was collapsed into 39 food groups previously used in other studies (S2 Table) [15,16]. For modelling meal-specific food intake, four eating occasions were chosen: breakfast, lunch, afternoon snack, and dinner, based on four observed peaks in food consumption (S2 Fig). Meal food intakes were analysed separately by meal type to identify foods that were consumed together. For modelling habitual food intake, we averaged all available 24hDRs per participant and all 11 eating occasions in the day were taken into account.

### Statistical methods and network analysis

GGMs describe conditional independence between variables, i.e., the relationship between two variables independent of the effect of other variables. They can be used to produce probabilistic graphs in which nodes represent variables and edges represent a relationship between the variables. These graphs can be quantified using partial correlations, under the assumption of a normal distribution. A high-dimensional multivariate data set can have no or few 0 values, which would form very dense, less informative graphical representations of the networks. For this reason, regularization methods for covariance estimation are available. Regularization is achieved by choosing a penalty parameter (λ >0), which reduces the variance and helps avoid overfitting of the model (avoiding the false inclusion of edges) [11]. Various methods are available for choosing the penalty parameter λ [17].

In this study, due to highly skewed data, the meal and habitual dietary networks were derived through SGCGMs, which is a nonparametric extension of GGMs. It performs the nonparanormal skeptic (Spearman/Kendall estimates preempt transformations to infer correlation) transformation in order to perform semiparametric analyses suited for highly skewed data [18,19]. This transformation is based on a nonparametric ranking of correlation coefficient estimators using Spearman’s rho and Kendall’s tau and offers an alternative for estimating high dimensional undirected graphical models without requiring normal distribution of the underlying data [20].

For the analyses here presented, skeptic transformed inverse covariance matrices were estimated using the “huge” R package [11,19]. The selection of the optimal penalization λ was performed with a tenfold cross-validated graphical lasso (glasso), which was run in R with the package “nethet” [21]. Communities, sets of closely related links, were detected within all identified networks to facilitate interpretation using the R package “linkcomm”, which is able to detect nested and overlapping communities in networks [22]. For food groups belonging to more than one community, centrality was assessed as a measure for the importance of a node based on the number of communities it belongs to [23]. The identified networks and corresponding communities were exported for formatting to CorelDRAW Graphics Suite X3 (Corel GmbH, Munich; www.corel.de). Food groups were considered to form a network when three or more groups were related to each other. Partial correlations equal or greater than ± 0.30 were considered as strong. The proportion of (direction-specific) relations (i.e. edges) from meal-specific networks present also in the habitual network was used as measure of the degree of appearance or reflection in the habitual network. All statistical analyses were performed in SAS (Version 9.4, Enterprise Guide 6.1, SAS Institute Inc., Cary, NC, USA) or R (Version 3.1.3, R Foundation for Statistical Computing, Vienna, Austria).

### Statement of previously published data

Previous publications have presented GGM dietary networks established from food frequency questionnaire (FFQ) data of the EPIC-Potsdam cohort collected at baseline between 1994 and 1998 (8, 20). In our analysis we used multiple 24hDRs from a subgroup collected in 2010–2012. Furthermore, the previous publications did not assess communities or centrality of food groups. Another publication is based on spearman correlation to understand PCA patterns, as such patterns are also based on correlations [4], while this analysis is based on GGM approach, i.e., using partial correlations to identify networks. These networks visualize combinations of food intake consumed at the meal level reflecting the intake patterns.

## Results

Baseline characteristics of all 814 participants are shown on Table 1. Participants were on average 65.5 years old, had a mean BMI of 27.5 kg/m^2^, and the majority was sedentary. A total of n = 2,411 breakfast observations (mean time 08:02), n = 2,236 lunch observations (mean time 12:37), n = 2,119 afternoon snack observations (mean time 15:31), and n = 2,346 dinner observations (mean time 18:45) were available. Mean intakes of the food groups per meal type and mean habitual intakes are shown on Table 2.

**Table 1:** Participants’ characteristics at the time of the first visit.

Characteristics	N = 814
Age, y	65.5 ± 8.4
Sex (%)	
Men	411 (50.5)
Women	403 (49.5)
BMI, kg/m^2^	27.5 ± 4.4
Physical activity level (ratio TEE/REE) (%)^1^	
Extremely inactive (< 1.4)	136 (19.9)
Sedentary (1.4 to < 1.7)	363 (53.0)
Moderately active (1.7 to < 2.0)	159 (23.2)
Vigorously active (2.0 to < 2.4)	25 (3.6)
Extremely active (≥ 2.4)	2 (0.3)
Education attainment (%)	
Currently in training/no certificate or skill	267 (32.8)
Professional school	187 (23.0)
≥ University	360 (44.2)
Smoking status (%)	
Never	377 (46.3)
Former	353 (43.4)
Smoker	84 (10.3)

Values are means ± SDs unless otherwise indicated.

^1^ *n* = 685; BMI, body mass index; TEE, total energy expenditure; REE, resting energy expenditure.

**Table 2:** Mean meal and habitual intake by food group.

Food group	Breakfast (g/meal) (n = 2,411)	Lunch (g/meal) (n = 2,236)	Afternoon snack (g/meal) (n = 2,119)	Dinner (g/meal) (n = 2,346)	Habitual (g/day) (n = 814)
Potatoes	0.01±0.40	72.53±92.30	1.48±16.39	12.46±44.80	81.7±66.5
Leafy vegetables	0.22±4.85	5.62±25.98	0.16±4.51	5.52±24.42	11.6±22.3
Fruiting & root vegetables	7.58±30.00	33.93±72.49	2.25±18.45	56.17±84.38	103±83.7
Cabbages	0.01±0.61	17.86±47.91	0.41±7.86	5.40±31.11	22.5±33.7
Other vegetables	0.32±4.55	23.91±58.39	0.58±6.80	9.74±34.98	32.9±38.6
Legumes	1.22±14.56	3.56±25.37	0.70±15.09	0.96±10.57	6.64±27.2
Fresh fruits	36.73±72.05	52.68±94.40	19.93±67.53	38.41±93.10	231±154
Nuts	0.69±4.60	0.23±2.64	0.27±5.24	0.24±2.74	3.95±10.2
Other fruits	1.22±18.03	3.99±28.93	0.72±14.16	2.21±25.07	10.2±33.2
Milk & dairy products	58.88±97.18	32.37±75.63	20.27±52.55	26.29±78.47	167±153
Cheese	13.08±20.13	3.28±12.82	0.67±5.92	18.27±26.56	37.4±27.1
Desserts	0.06±2.80	9.23±39.27	3.73±23.74	1.85±17.46	17.6±33.8
Pasta & rice	0.31±5.75	17.84±55.32	0.54±10.86	4.81±31.07	23.1±39.4
Bread	52.09±33.41	10.47±24.39	3.51±13.66	41.16±35.54	113±48.4
Breakfast cereals	2.53±11.73	0.26±3.78	0.13±3.06	0.22±4.40	3.40±12.1
Other cereals	1.27±7.24	1.06±7.49	0.19±2.54	1.31±10.88	5.30±11.9
Red meat	0.89±10.47	27.46±56.58	1.42±16.90	11.19±39.99	39.5±46.3
Poultry	0.18±4.68	9.15±35.84	0.35±7.52	5.67±29.96	14.8±27.4
Processed meat	9.78±18.12	20.96±44.27	2.32±15.96	25.08±37.36	60.8±46.1
Fish	1.69±10.66	11.37±43.05	0.57±9.68	11.25±40.82	24.1±37.9
Eggs	10.16±24.14	4.79±19.53	0.37±5.76	2.98±15.40	18.7±22.3
Margarine	5.20±9.87	2.18±5.94	0.33±2.43	4.95±9.43	13.2±16.9
Vegetable oils	0.22±2.07	2.84±7.33	0.10±1.42	1.98±6.09	5.06±6.36
Butter & animal fat	7.78±11.32	3.16±7.67	0.50±3.13	5.64±10.99	17.6±18.5
Sugar & confectionery	18.96±22.32	2.16±9.11	3.71±11.78	2.06±8.07	38.0±29.7
Cakes & cookies	1.96±17.19	4.23±30.15	51.31±72.47	1.56±14.50	59.2±55.5
Fruit & vegetable juices	14.69±49.74	16.14±56.78	6.50±36.50	14.58±53.63	94.5±144
Soft drinks	0.57±13.16	7.82±47.78	4.17±35.78	13.14±71.60	48.1±126
Tea	84.94±172.94	24.62±86.50	34.61±103.49	90.52±157.37	355. ±381
Coffee	220.36±170.34	17.99±63.07	152.87±140.45	3.59±31.03	447±230
Water	28.53±71.62	92.74±127.49	59.84±121.59	80.63±130.08	740±477
Wine	0.45±10.53	5.89±40.38	3.15±25.82	12.10±56.78	57.3±101
Beer	0±0	14.76±78.08	7.35±60.70	56.62±164.54	173. ±316
Spirits	0±0	0.02±0.67	0.12±2.08	0.13±2.40	1.59±6.99
Other alcoholic beverages	0±0	0.65±12.34	0.80±13.99	0.64±9.78	4.99±20.1
Sauces	0.40±2.67	17.26±37.74	0.45±4.69	6.72±19.74	24.2±25.1
Condiments	0.33±1.47	0.91±3.47	0.17±1.46	1.10±3.80	2.79±4.60
Soups	2.29±24.40	36.20±93.40	1.51±19.88	11.99±57.41	51.8±74.8
Snacks	0.18±2.66	0.59±9.94	0.12±2.72	0.68±9.12	1.60±8.71

Values are means ± SDs.

### Breakfast networks

**Fig 1:**
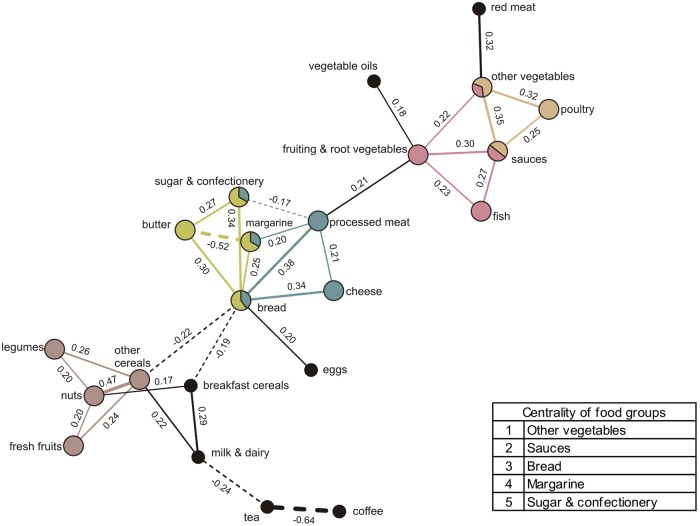
Dietary meal network and communities derived from breakfast intakes (n = 2,411) by Gaussian graphical models. Nodes represent food groups. Edges represent conditional dependencies between food groups revealed by partial correlation coefficients. The absence of an edge between 2 food groups indicates conditional independence between them. Continuous edges show positive partial correlations while broken edges show negative partial correlations. Line thickness is proportional to the strength of the correlations between food groups. Communities are represented by matching node and edge colours. Black nodes correspond to food groups not assigned to a community. Centrality indicates importance of a food group based on the number of communities it belongs to.

The SGCGM analysis identified one major breakfast network (Fig 1) where foods are grouped into five communities. Starting in the lower left, a community is made up of fresh fruits, nuts, legumes, and other cereals, linked by positive correlations, among which the strongest is nuts and other cereals (partial correlation = 0.47). Next, two partly overlapping communities can be observed; one composed of bread consumed together either with margarine (partial correlation = 0.25), or with butter and sugar & confectionery (partial correlations = 0.30 and 0.34, respectively) and the other composed of bread consumed with processed meat and cheese (partial correlations = 0.38 and 0.34, respectively). Processed meat in turn is consumed with margarine (partial correlation = 0.20) but not with sugar & confectionery (partial correlation = -0.17). The fourth and fifth communities found are also overlapping and they describe the dependency structure of intake of sauces, fish, fruiting & root vegetables, other vegetables, and poultry. Central food groups were in decreasing importance as follows: other vegetables, sauces, bread, margarine, and sugar & confectionery. Not all food groups that are part of this network were represented in the communities; tea and coffee, for instance, are strongly correlated with each other (partial correlation = -0.64), but were not part of a community, suggesting that these food groups are less closely linked to other food groups in the network (Fig 1).

### Lunch networks

**Fig 2:**
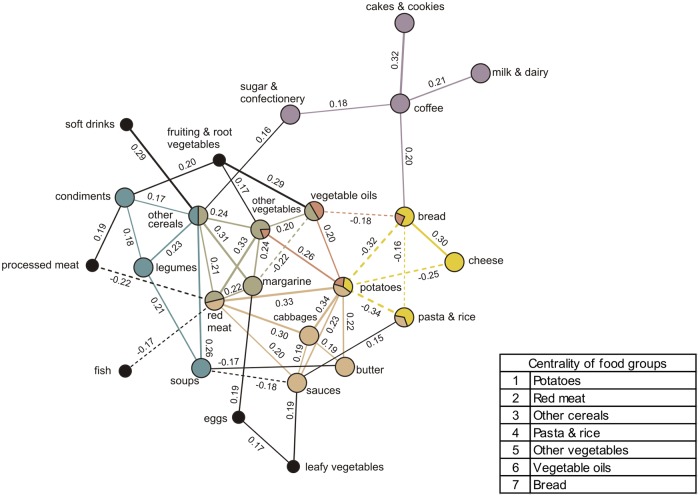
Dietary meal network and communities derived from lunch intakes (n = 2,236) by Gaussian graphical models. Nodes represent food groups. Edges represent conditional dependencies between food groups revealed by partial correlation coefficients. The absence of an edge between 2 food groups indicates conditional independence between them. Continuous edges show positive partial correlations while broken edges show negative partial correlations. Line thickness is proportional to the strength of the correlations between food groups. Communities are represented by matching node and edge colours. Black nodes correspond to food groups not assigned to a community. Centrality indicates importance of a food group based on the number of communities it belongs to.

Our analysis identified one major lunch network for this meal characterized by six communities (Fig 2). Overall, with a more complex structure, this network reflects a variable consumption of foods. The community on the left describes the dependency structure between other cereals, condiments, legumes, and soups. In the centre of the network, there is a community composed by other cereals, other vegetables, vegetable oils, margarine, and red meat, with a strong positive correlation between red meat and other vegetables (partial correlation = 0.33) and with a negative correlation between margarine and vegetable oils (partial correlation = -0.22). A partially overlapping community describes the dependency structure between other vegetables, vegetable oils, bread, and potatoes. Next, on the right side of the network a community was detected where bread correlates strongly positively with cheese (partial correlation = 0.30) and negatively with potatoes and pasta & rice (partial correlations = -0.32 and -0.16, respectively), and potatoes correlate negatively with cheese and pasta & rice (partial correlations = -0.25 and -0.34, respectively). At the bottom of the lunch network, a community with only positive correlations including potatoes, cabbages, red meat, sauces, butter, and pasta & rice is shown. The edges linking cabbages–potatoes–red meat–cabbages show strong correlations (partial correlations = 0.34, 0.33, 0.30, respectively). Finally, the top right of the network shows a community of sweet foods composed of coffee consumed together with either cakes & cookies, milk & dairy, or sugar & confectionery (partial correlations = 0.32, 0.21, 0.18, respectively). Foods with central roles (pertaining to more than one community) were in decreasing importance as follows: potatoes, red meat, other cereals, pasta & rice, other vegetables, vegetable oils, and bread. A few food groups were represented in the lunch network but were not part of any community, such as soft drinks, fruiting & root vegetables, processed meat, fish, eggs, and leafy vegetables (Fig 2).

### Afternoon snack networks

**Fig 3:**
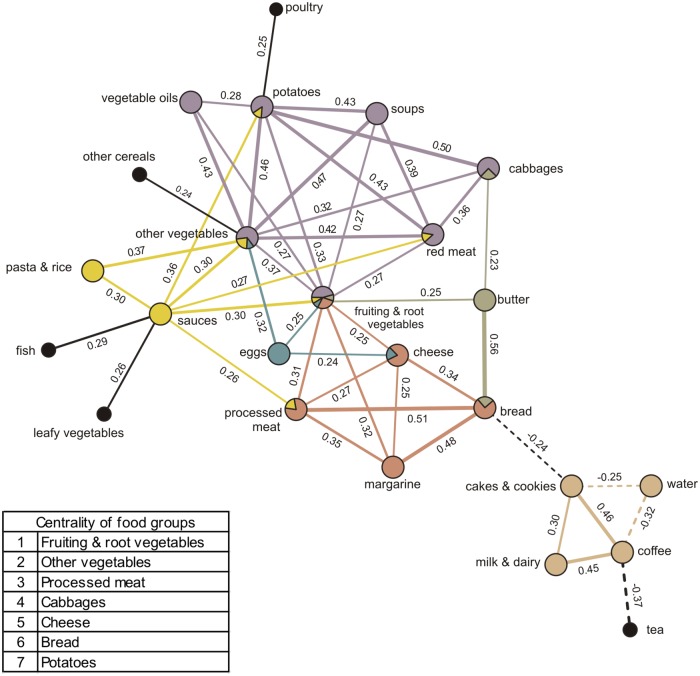
Dietary meal network and communities derived from afternoon snack intakes (n = 2,119) by Gaussian graphical models. Nodes represent food groups. Edges represent conditional dependencies between food groups revealed by partial correlation coefficients. The absence of an edge between 2 food groups indicates conditional independence between them. Continuous edges show positive partial correlations while broken edges show negative partial correlations. Line thickness is proportional to the strength of the correlations between food groups. Communities are represented by matching node and edge colours. Black nodes correspond to food groups not assigned to a community. Centrality indicates importance of a food group based on the number of communities it belongs to.

There was one afternoon snack network identified with six communities (Fig 3). Similar to the lunch network, this network reflects a variable food intake, though it revealed stronger partial correlations among intakes. At the bottom of the network, a community was identified where coffee, cakes & cookies, and milk & dairy correlate strongly positively with each other (partial correlations = 0.46, 0.30, 0.45, respectively) and water correlates negatively with coffee and cakes & cookies (partial correlations = -0.32, -0.25, respectively). This community is linked with the two following communities through a negative correlation between cakes & cookies and bread. Bread, on one side, belongs to a community where bread is consumed with margarine, processed meat, and cheese and where fruiting & root vegetables are consumed with processed meat, margarine, and cheese. On the other side, bread belongs as well to a community where it is consumed with butter (partial correlation = 0.56) and butter consumed with cabbages and with fruiting & root vegetables. The largest community within this network involved potatoes, vegetable oils, other vegetables, fruiting & root vegetables, red meat, cabbages, and soups. Central food groups were, with decreasing order of importance: fruiting & root vegetable (part of five different communities), other vegetables, processed meat, cabbages, cheese, bread, and potatoes. Only tea, fish, leafy vegetables, other cereals, and poultry were part of the network but did not belong to any community (Fig 3).

### Dinner networks

**Fig 4:**
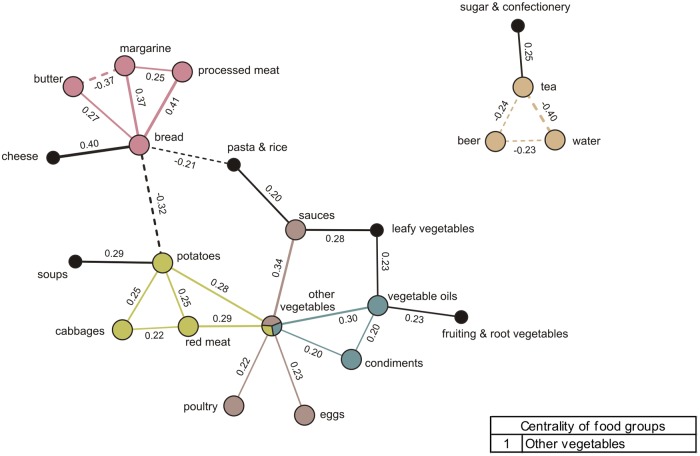
Dietary meal networks and communities derived from dinner intakes (n = 2,346) by Gaussian graphical models. Nodes represent food groups. Edges represent conditional dependencies between food groups revealed by partial correlation coefficients. The absence of an edge between 2 food groups indicates conditional independence between them. Continuous edges show positive partial correlations while broken edges show negative partial correlations. Line thickness is proportional to the strength of the correlations between food groups. Communities are represented by matching node and edge colours. Black nodes correspond to food groups not assigned to a community. Centrality indicates importance of a food group based on the number of communities it belongs to.

The SGCGM analysis identified one major dinner network and a smaller network (Fig 4). The major network shows a complex meal composition with four communities and one central food group, other vegetables, belonging to three communities. On the top right, a community shows that bread is consumed with processed meat and with either margarine or butter. Bread correlated strongly positive with processed meat and margarine (partial correlations = 0.41 and 0.37, respectively) and butter and margarine correlated strongly negatively (partial correlation = -0.37). Another community shows the concomitant consumption of potatoes with cabbages, red meat, and other vegetables. On the upper right, an independent community was found in a smaller dinner network. This final community is composed by beer, tea, and water, which all correlate negatively with each other, indicating that only one of these beverages is chosen in this meal. Sugar & confectionery was also part of this network, correlating positively with tea, but it was not present in any community. Other food groups such as cheese, soups, pasta & rice, leafy vegetables, and fruiting & root vegetables were also part of the larger dinner network but did not form part of a community, suggesting these links are less closely linked to other food groups in the network (Fig 4).

### Habitual diet network

**Fig 5:**
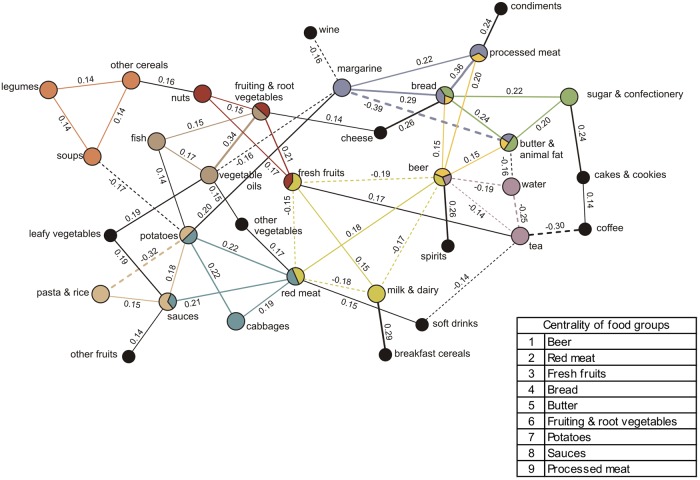
Dietary meal network and communities derived from participants’ habitual daily intake (n = 814) by Gaussian graphical models. Nodes represent food groups. Edges represent conditional dependencies between food groups revealed by partial correlation coefficients. The absence of an edge between 2 food groups indicates conditional independence between them. Continuous edges show positive partial correlations while broken edges show negative partial correlations. Line thickness is proportional to the strength of the correlations between food groups. Communities are represented by matching node and edge colours. Black nodes correspond to food groups not assigned to a community. Centrality indicates importance of a food group based on the number of communities it belongs to.

One habitual network was identified by SGCGMs (Fig 5). This network is formed by a complex structure of interrelated food groups, where beer, red meat, fresh fruits, bread, butter, fruiting & root vegetables, potatoes, sauces, and processed meat play central roles, with decreasing importance. Overall, the ten communities identified within this network show: i) positive correlations between legumes, other cereals, and soups; ii) positive correlations between nuts, fruiting & root vegetables, and fresh fruits; iii) positive correlations between fish, fruiting & root vegetables, and vegetable oils; iv) positive correlations between sauces and pasta & rice and with potatoes but a negative correlation between potatoes and pasta & rice; v) positive correlations between cabbages, potatoes, red meat, and sauces; vi) a positive correlation between fresh fruits and milk & dairy as well as between red meat and beer, while fresh fruits and milk & dairy correlated negatively with red meat and beer; vii) positive correlations of beer with bread, processed meat, and butter; viii) negative correlations between beer, water, and tea; ix) positive correlations between bread, butter, and sugar & confectionery; and x) positive correlations between bread, margarine, and processed meat, and a negative correlation between margarine and butter. Out of the 39 food groups, 33 of them were part of this complex network and 22 of them formed part of at least one community. Soft drinks and wine formed part of this network but did not show in any of the meal networks.

### Comparison of meal and habitual dietary networks

In general, partial correlations were stronger on the meal-specific dietary networks than on the habitual dietary network, especially in the case of the afternoon snacks. Some food groups that had central roles in meal networks were also central food groups in the habitual network, such as bread and potatoes. Four of the ten communities in the habitual network resembled communities found in the meals: the community formed by beer, water, and tea was also found in dinner; the community formed by bread, processed meat, margarine, and butter was similar to one seen in dinner; the community formed by soups, legumes, and other cereals was similar to one observed in lunch; and the community formed by red meat, cabbages, potatoes, and sauces was part of a larger community found in lunch. A few food groups that showed strong partial correlations only in a specific meal persisted on the habitual network, such as the relationship between milk & dairy and breakfast cereals seen in the breakfast network. In general, correlations between food groups were in the same direction (positive or negative) in meal and habitual networks, with the exception of soups and potatoes, which was positive in the afternoon snack and dinner networks and negative in the habitual network. By estimating the percentage of connections between foods in the meal-specific networks that were also present in the habitual dietary network we found that the dinner network was best reflected in the habitual network. Specifically, we found 50.0% of the breakfast, 36.2% of the lunch, 33.3% of the afternoon snack, and 64.3% of the dinner networks relations between food groups were present in the habitual network (**Figures A-D in S3 File**). On the other hand, 34% of the relations seen in the habitual network were not present in any of the meal-specific networks (**Figure E in S3 File**).

## Discussion

This study identified meal dietary networks through SGCGMs, an extension of GGMs suited for non-normal distributed data [20]. Communities and centrality of food groups were detected to assist interpretation. GGMs had not yet been used for meal-specific analyses. The meal-specific networks showed clear differences in composition and strength of correlations. The combination of bread, cheese, processed meat, and margarine or butter was present in most networks (all meals except for lunch). Afternoon snack, which is a smaller but a culturally important eating occasion in Germany (equivalent to the British *tea time*) showed the strongest correlations, where the communities including bread correlated negatively with coffee, milk & dairy, and cakes & cookies, suggesting that either one or other combination is consumed during this meal. The networks for lunch and afternoon snack showed a complex structure, indicating a more variable food intake. Potatoes, red meat, other vegetables, and bread were often central food groups in the dietary networks but differences for each meal were evident. Out of the four main meals, dinner networks were best reflected in the habitual dietary network and the afternoon network was the least reflected despite the strong partial correlations. Despite a variable food intake in dinner, this network was among the least dense. The variable and substantial intake in this meal may have contributed to the better representation in the habitual dietary network. This analysis revealed different strengths of correlation and combinations of food intake at meal and habitual intake levels. As food is consumed at meal level, habitual level intake may not reflect the clear picture of food intake patterns in a population.

The interrelation between intakes of different food groups is complex and when analysed using meal-aggregated data such as FFQs, weaker correlation structures are observed, which may arise due to an increased intra-subject variability [24]. This is usually a phenomenon seen in building exploratory dietary patterns, such as PCA-patterns. In our study, habitual diet takes into account some day-to-day variation within study participants, while the meal intakes were analysed independently from participant to accurately reflect foods eaten together. This resulted in stronger partial correlations in the meal networks. However, some characteristics of the habitual diet that were not traceable to the meal networks might come from other factors, such as individual characteristics and preferences and eating occasions not considered in this analysis such as smaller snacks [25,26]. Further investigations are required to understand why some of the relationships among food groups at the habitual level did not appear at the meal level.

GGMs were previously applied to dietary data from the EPIC-Potsdam cohort [10], a population of which our participants are a sub-cohort. In this study data from FFQs were used. Therefore, although the results highlight an overall-diet structure, they do not represent meal level intake-specific relationships. Despite numerous methodological differences in the dietary intake assessment and pattern analysis (food networks) between this and our study, we could observe certain similarities between their dietary networks and our habitual dietary network; for example, potatoes and red meat, which often played central roles in our meal networks, were simultaneously linked to multiple food groups in the principal networks. Nevertheless, in order to understand dietary habits, which in turn are the drivers of dietary patterns, food consumption should be analysed in a timing-, or meal-specific manner [27].

Established meal-specific habits are known to be present in the human diet. For instance, a few studies have observed a more homogeneous and simple composition of early meals and a more complex and varied composition of later meals [28,29]. Meal setting is an important factor affecting meal composition. For example, breakfast is more likely to be consumed at home and dinner outside of home [30]. Although we did not explore meal setting in our study, we did see a more simple structure of the breakfast network and a dinner network that was closest to the habitual network. In line with these observations, our recent study comparing meal and day level food intake to habitual diet using the same study sample revealed a consistent composition of the breakfast meal and a more variable intake at dinner, which was the meal that contributed the most to the formation of PCA-habitual dietary patterns [4]. Such differences across meals and similarities within them are not visible in day-aggregated data such as data commonly used to derive dietary patterns.

Commonly, the method of choice for deriving dietary patterns is PCA. This method (PCA) was previously applied to this study sample to derive breakfast patterns [31]. As PCA is also based on correlations, two PCA patterns, i.e., *processed food pattern* and *dairy & cereal pattern*, shared considerable similarity with our breakfast network. However, GGMs identify sparse networks reflecting patterns of intake and visualize the identified combinations of intakes in relation to each other. Smaller sub-networks (communities) are easier to interpret as compared to PCA patterns, which comprise of all the food variables [22], and GGM networks (specifically residualized, or conditional independence networks) have the ability of showing conditional independence between food groups [9] which PCAs or simple correlation analyses do not.

Furthermore, consistent intakes are underrepresented in PCA patterns [4] but this was not the case in GGM dietary networks. Therefore, GGMs are a valuable tool for the analysis and interpretation of the complex data structure of food intakes often seen in the field of nutritional epidemiology. To our knowledge, these tools had not yet been applied to dietary networks in the context of meals. Other exploratory methods have been used to explore meal patterns. For example, Hearty et al. [32] used artificial neural networks and decision trees with the purpose of predicting dietary quality in terms of the Healthy Eating Index. These are complicated but interesting applications of predictive machine learning models that may provide a better understanding of how hypothesis-based dietary patterns (i.e., dietary quality indices) [33] arise in a population but not directly comparable to the here presented data-driven GGM dietary networks, which are not intended to identify meal patterns predictive of or meeting dietary guidelines, but are rather describing the intake of the studied population in detail. Nevertheless, GGM dietary networks can also be combined with methods to predict disease or adherence to dietary guidelines similar to how is done with PCA patterns [33]. Such procedure is exemplified in the recent publication by Iqbal et al. [34] using habitual (non-meal specific) GGM dietary networks. Overall, hypothesis-based or data-driven methods should be considered based on the research question of interest [1].

Other studies estimated habitual diet with more sophisticated statistical methods such as the National Cancer Institute (NCI) method [35–37]. This method adjusted out day-to-day variation by accounting for food intake in the analysed population. In the present study we could not apply the NCI method due to a very high proportion of zeros at meals resulting in convergence problems of the statistical algorithms. Furthermore, our methods remain consistent with our previous work on meal, day, and habitual intake analyses [4]. Working with non-normal data implies some limitations; in order to circumvent the Gaussian assumption, SGCGMs perform a rank-based transformation of the original variables. Estimates, power, and Type I error can be dependent of the transformation method, sample size, and degree of non-normality [38,39]. Nevertheless, our sample size was large, with at least 2,119 observations per meal; also, for the descriptive purpose of this study, potential alterations in power and Type I error play a less important role. Nevertheless, this should be kept in mind for studies intending to find diet-disease associations. The stability of the resulting networks depends also from the approach used, which could be threshold- or model-based. Threshold-based networks, also called relevance networks, remove correlations weaker than a pre-determined correlation strength [40]. However, this threshold is typically arbitrary and may result in inclusion of false edges or exclusion of true edges [41]. In this study, we preferred a model-based approach (lasso using cross-validation), which seeks to identify a sparse model (identifying only important variables) by maximizing log-likelihood of the data [42,43].

In conclusion, SGCGMs identified meal-specific dietary networks describing combinations of foods that are eaten together. Clear differences were seen across meals. The habitual dietary network retained some but not all information from the meal-specific dietary networks and additionally showed relations not present at the meal level. As a result, interpretation of such habitual networks needs to be done carefully. Analysing food intake using both meal-based and habitual intake data can provide a broader picture about eating behaviour than using one approach only. GGMs and SGCGMs can be used as tools to obtain meal-specific insights of diet, which may be used as a foundation for meal-based recommendations. Nevertheless, these are methods that have not been applied often in the field of nutritional epidemiology and warrant further applications in other populations due to their specific features.

## Supporting information

S1 Table List of all eating occasions with participant-identified labels used to record food intake in the 24-hour dietary recalls. (DOCX)

S2 Table List of 39 food groups used throughout the analyses. (DOCX)

S1 Fig Flow-chart of participants of the validation sub-study within the EPIC Potsdam cohort. (DOCX)

S2 Fig Mean contribution (% amount in grams) of eating occasions to food consumption over the day (n = 814). (DOCX)

S3 Fig Meal networks emphasizing relations also present in the habitual network (S3 Figs A-D) and habitual network emphasizing relations not found in any of the meal-specific dietary networks (S3 Fig E). (DOCX)

## Abbreviations

24hDR: 24-hour dietary recalls
BMI: Body Mass Index
EPIC: European Prospective Investigation into Cancer and Nutrition
FFQ: food frequency questionnaire
GGM: Gaussian Graphical Model
NCI: National Cancer Institute
PCA: principal component analysis
SGCGM: Semiparametric Gaussian Copula Graphical Model
