# Supplementary material for: Meal and habitual dietary networks identified through Semiparametric Gaussian Copula Graphical Models in a German adult population
Source: PLoS One. 2018 Aug 24;13(8):e0202936. doi: 10.1371/journal.pone.0202936 (PMC6108519; doi:10.1371/journal.pone.0202936)
Supplement: S1 Table — (DOCX) [file pone.0202936.s001.docx]

S1 Table: Eating occasions with participant-identified labels used to record food intake in the 24-hour dietary recalls

| Eating occasion No. | Eating occasion participant-identified label |
| --- | --- |
| 1 | Before breakfast |
| 2 | Breakfast |
| 3 | During morning |
| 4 | Before lunch |
| 5 | Lunch |
| 6 | After lunch |
| 7 | During afternoon (afternoon snack) |
| 8 | Before dinner |
| 9 | Dinner |
| 10 | After dinner |
| 11 | During evening |
