# Supplementary material for: Meal and habitual dietary networks identified through Semiparametric Gaussian Copula Graphical Models in a German adult population
Source: PLoS One. 2018 Aug 24;13(8):e0202936. doi: 10.1371/journal.pone.0202936 (PMC6108519; doi:10.1371/journal.pone.0202936)
Supplement: S2 Table — (DOCX) [file pone.0202936.s002.docx]

S2 Table: List of 39 food groups used throughout the analyses^1^

| Food group code | Name | Description |
| --- | --- | --- |
| 1 | Potatoes | Potatoes and sweet potatoes |
| 2 | Leafy vegetables | Leafy green vegetables |
| 3 | Fruiting and root vegetables | Fruiting vegetables: artichoke, avocado, eggplant, green beans, chili peppers, cucumber, bell peppers, tomatoes;  Root vegetables: kohlrabi (German turnip), manioc, radish, beets, carrots |
| 4 | Cabbages | Broccoli, cauliflower, cabbage, sauerkraut |
| 5 | Other vegetables | Mushrooms, peas, corn, sprouts, garlic, onions, stalk vegetables (fennel, leek, celery, asparagus, bamboo shoots), vegetable mixes |
| 6 | Legumes | White beans, kidney beans, black beans, other beans, chick peas, lentils |
| 7 | Fresh fruits | All fresh fruits |
| 8 | Nuts | All nuts and seeds |
| 9 | Other fruits | Fruit mixes (for example in salads), dried fruits, canned fruits, olives |
| 10 | Milk and dairy products | Milk, dairy beverages, yogurt, eggnog, quark, cream (dairy and non-dairy based), creamer |
| 11 | Cheese | All cheeses |
| 12 | Desserts | Mousse, pudding, cream desserts, milk and water based ice creams and sorbets |
| 13 | Pasta, rice | All pasta and rice |
| 14 | Bread | White bread, whole grain bread, crackers and breadcrumbs |
| 15 | Breakfast cereals | All breakfast cereals with exception of muesli and oatmeal |
| 16 | Other cereals | Cornstarch, dough, yeast dough, pretzel sticks, oatmeal flakes, whole grain rye flakes, wheat flour, spelt flour, chips (potato and from other cereals), dumplings, bread sticks |
| 17 | Red meat | Non-processed meats from: beef, veal, pork, lamb, rabbit, other red meat (kangaroo) |
| 18 | Poultry | Non-processed meats from: chicken, turkey, duck, goose |
| 19 | Processed meat | Meatballs, cured ham and meat breast, salami and sausages, bacon, meatloaf, corned beef, mortadella, liver pate, meat jelly |
| 20 | Fish | All fish and shellfish |
| 21 | Eggs | Whole egg, egg whites, and yolks |
| 22 | Margarine | Margarine |
| 23 | Vegetable oils | Vegetable oils |
| 24 | Butter and other animal fat | Butter, lard |
| 25 | Sugar and confectionery | Honey, syrups, sugar, marmalades and jams, chocolate, chocolate bars and candies, candies, caramelized fruits and nuts, marzipan, chewing gum, licorice candy |
| 26 | Cakes and cookies | Cakes, pastries, cookies, pancakes and waffles |
| 27 | Fruit and vegetable juices | All fruit and vegetable juices |
| 28 | Soft drinks | Carbonated and non-carbonated soft drinks, alcohol free beer, tonic water, coconut milk |
| 29 | Tea | Black tea, green tea, herbal and fruit infusions |
| 30 | Coffee | Coffee and coffee substitute drinks |
| 31 | Water | Water (sparkling and still) |
| 32 | Wine | All wines containing alcohol |
| 33 | Beer | All beer containing alcohol |
| 34 | Spirits | Vodka, whiskey, rum, cognac, gin, other spirits |
| 35 | Other alcoholic beverages | Punch, herbal liquors, mulled wine, sparkling wine, egg liquor, sherry, ouzo, Campari, martini, amaretto, cherries in alcohol, baileys, other alcoholic beverages |
| 36 | Sauces | Tomato-based sauces, dips and dressings, mayonnaise-based sauces, dessert sauces, other sauces |
| 37 | Condiments | Vinegar, mustard, herbs, salt, pepper, artificial sweetener |
| 38 | Soups | All soups and broths |
| 39 | Snacks | Bread snacks (gratin, tomato and cheese), puff pastries with fillings, spring rolls, breaded or fried vegetables, spreads, vegetarian sausages |

^1^ Based on one of the pre-defined food groupings from the EPIC-Potsdam Study
