# Supplementary material for: Meal and habitual dietary networks identified through Semiparametric Gaussian Copula Graphical Models in a German adult population
Source: PLoS One. 2018 Aug 24;13(8):e0202936. doi: 10.1371/journal.pone.0202936 (PMC6108519; doi:10.1371/journal.pone.0202936)
Supplement: S1 Fig — (DOCX) [file pone.0202936.s003.docx]

Potentially eligible sample

23,881 active EPIC-Potsdam follow-up participants

1447 invitations sent out

(age and sex stratified random draw)

815 participated in the sub-study

(56% participation rate)

896 individuals

Three 24hDRs

n = 806

896 individuals

Two 24hDRs

n = 5

896 individuals

One 24hDRs

n = 3

896 individuals

Final study sample (n=814)

1 participant with dementia excluded

S1 Fig: Flow-chart of participants of a validation and calibration sub-study within the EPIC Potsdam cohort
