# Supplementary figures and images for: Meal and habitual dietary networks identified through Semiparametric Gaussian Copula Graphical Models in a German adult population
Source: PLoS One. 2018 Aug 24;13(8):e0202936. doi: 10.1371/journal.pone.0202936 (PMC6108519; doi:10.1371/journal.pone.0202936)

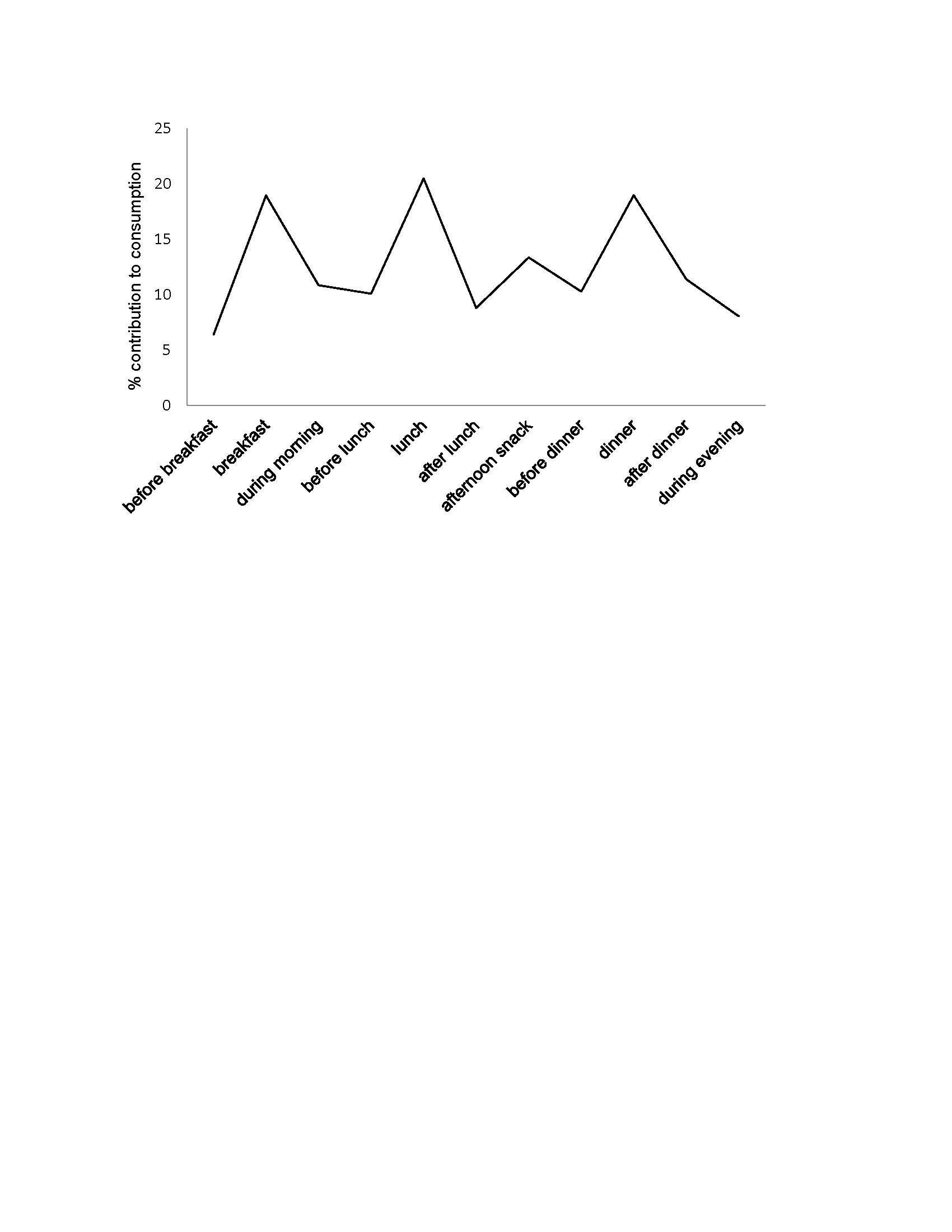


S2 Fig: Mean contribution (% amount in grams) of eating occasions to food consumption over the day (n=814)

Supplement: S2 Fig — (DOCX) [file pone.0202936.s004.docx]
