# Supplementary material for: Meal and habitual dietary networks identified through Semiparametric Gaussian Copula Graphical Models in a German adult population
Source: PLoS One. 2018 Aug 24;13(8):e0202936. doi: 10.1371/journal.pone.0202936 (PMC6108519; doi:10.1371/journal.pone.0202936)
Supplement: S3 Fig — (DOCX) [file pone.0202936.s005.docx]

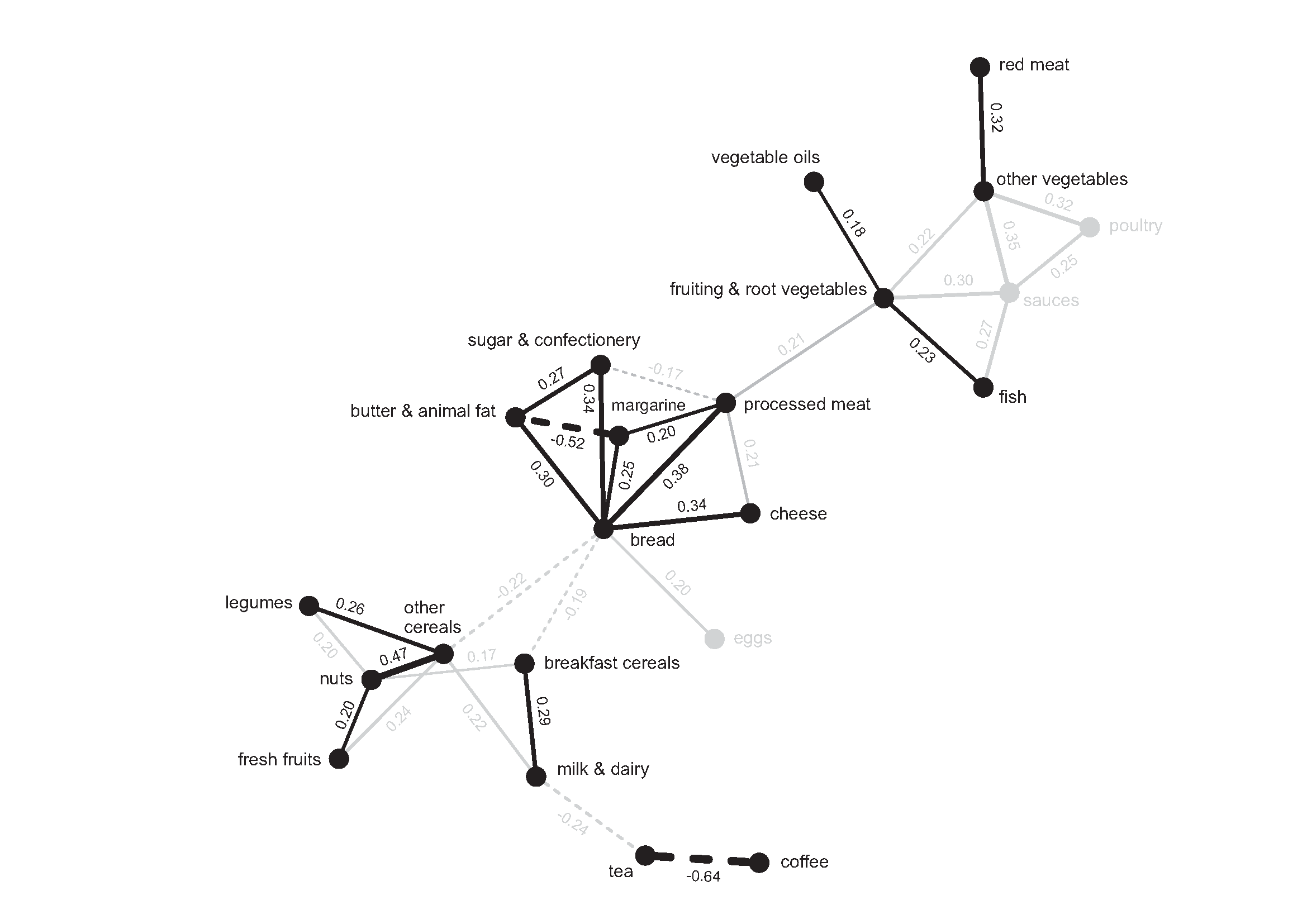


S3 Fig A: Breakfast network emphasizing relations also present in the habitual network. 50.0% of relations were present in the habitual network. Edges represent conditional dependencies between food groups revealed by partial correlation coefficients. The absence of an edge between 2 food groups indicates conditional independence between them. Continuous edges show positive partial correlations while broken edges show negative partial correlations. Line thickness is proportional to the strength of the correlations between food groups.


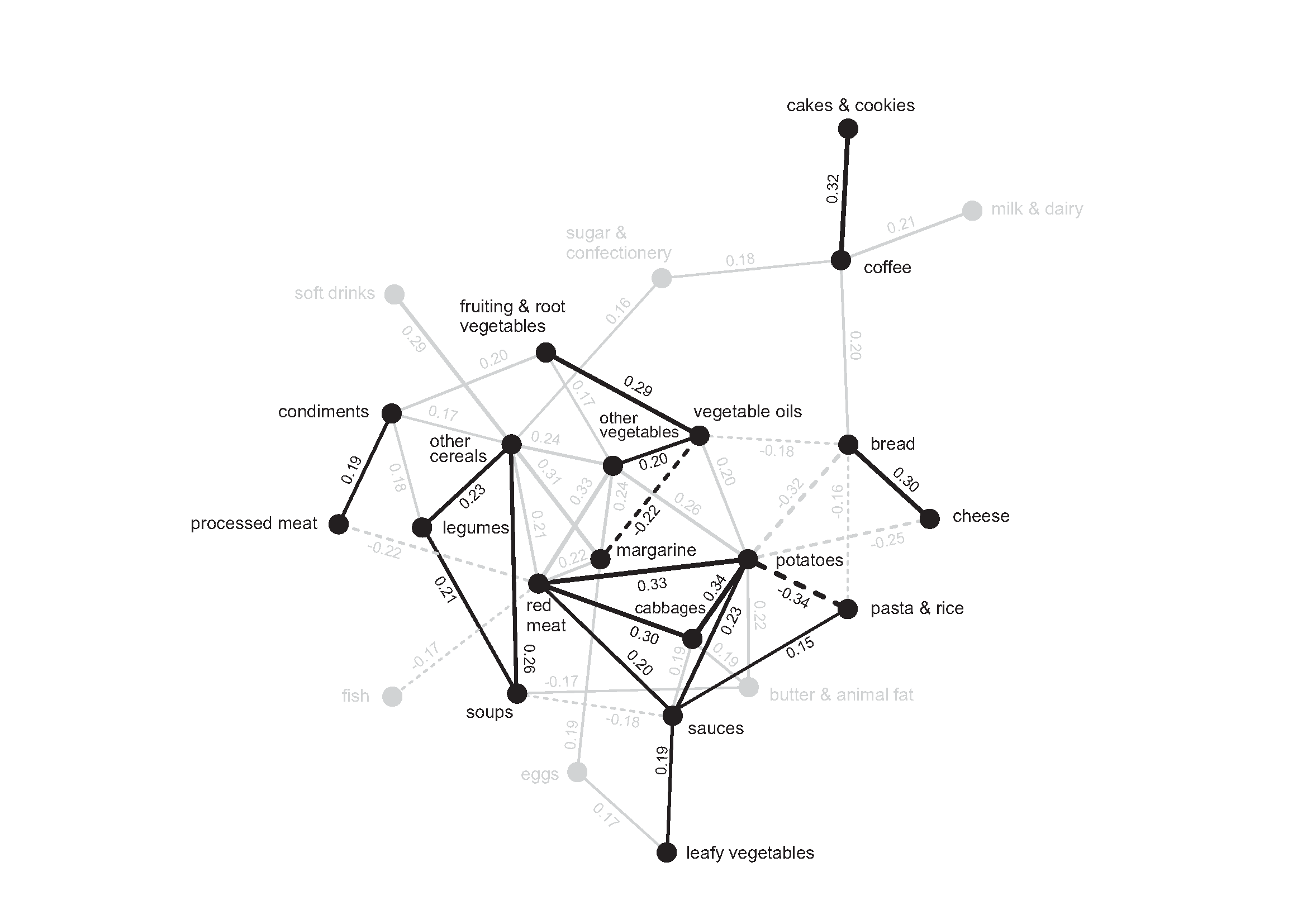


S3 Fig B: Lunch network emphasizing relations also present in the habitual network. 36.2% of relations were present in the habitual network. Edges represent conditional dependencies between food groups revealed by partial correlation coefficients. The absence of an edge between 2 food groups indicates conditional independence between them. Continuous edges show positive partial correlations while broken edges show negative partial correlations. Line thickness is proportional to the strength of the correlations between food groups.


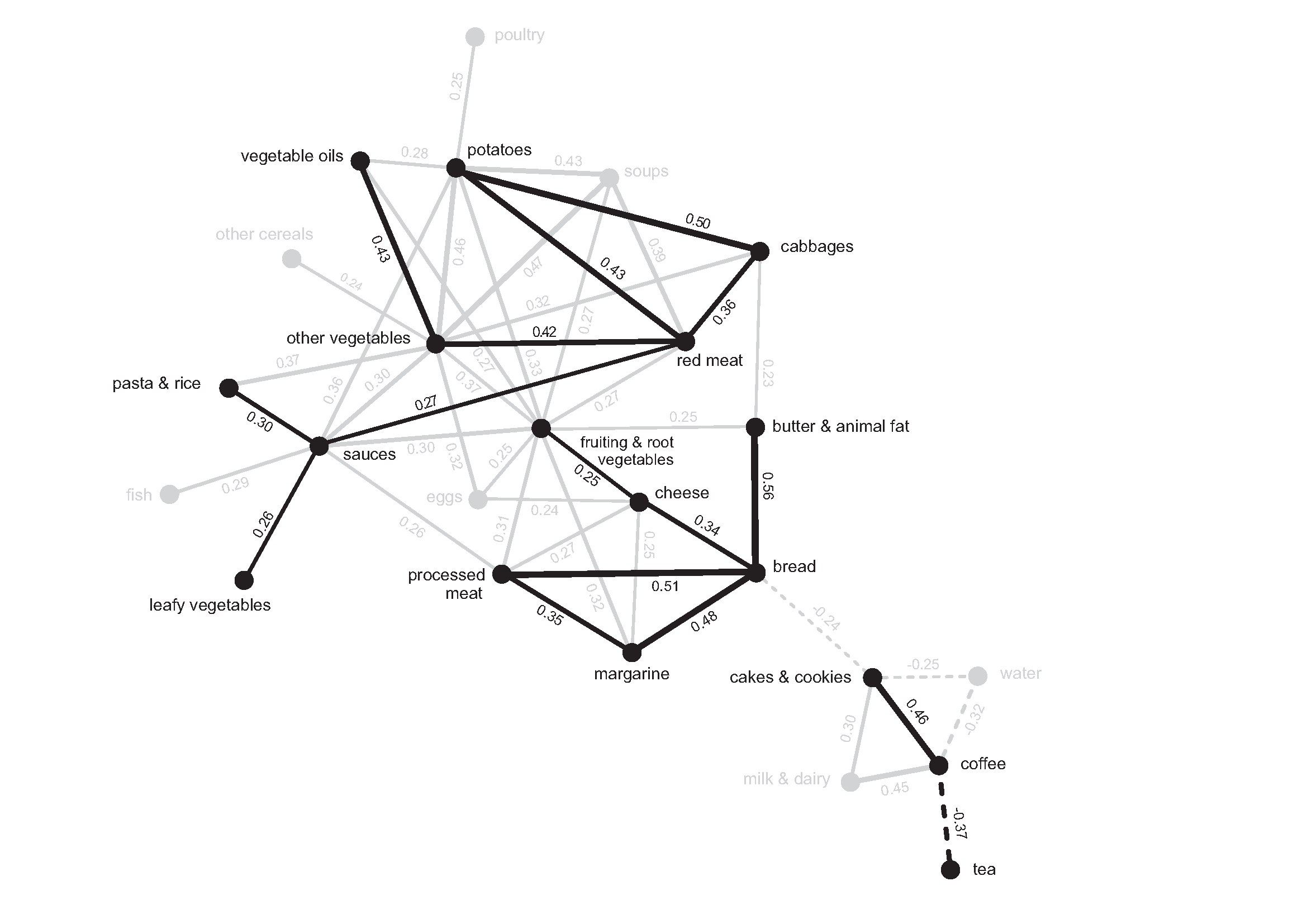


S3 Fig C: Afternoon snack network emphasizing relations also present in the habitual network. 33.3% of relations were present in the habitual network. Edges represent conditional dependencies between food groups revealed by partial correlation coefficients. The absence of an edge between 2 food groups indicates conditional independence between them. Continuous edges show positive partial correlations while broken edges show negative partial correlations. Line thickness is proportional to the strength of the correlations between food groups.


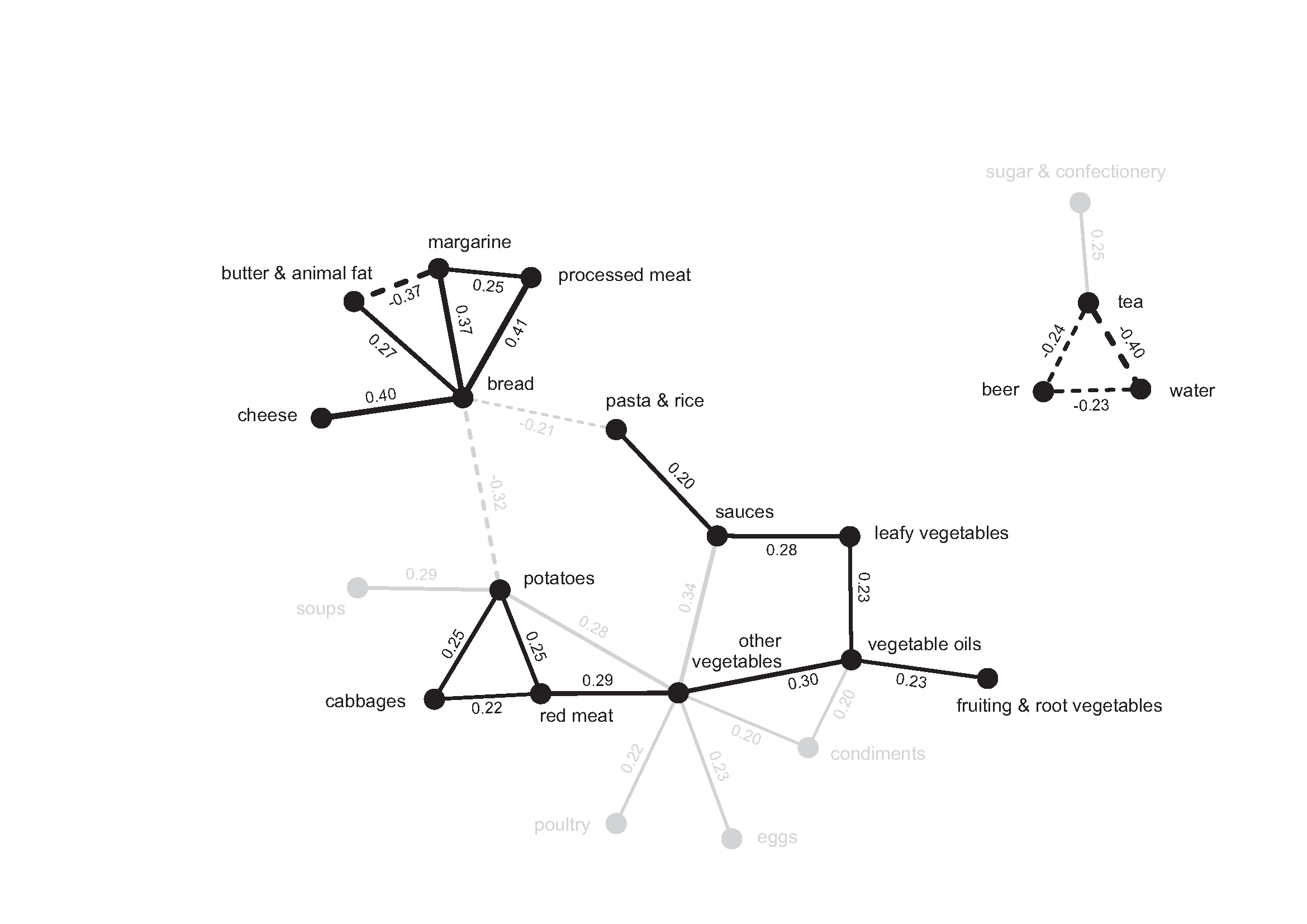


S3 Fig D: Dinner networks emphasizing relations also present in the habitual network. 64.3% of relations were present in the habitual network. Edges represent conditional dependencies between food groups revealed by partial correlation coefficients. The absence of an edge between 2 food groups indicates conditional independence between them. Continuous edges show positive partial correlations while broken edges show negative partial correlations. Line thickness is proportional to the strength of the correlations between food groups.


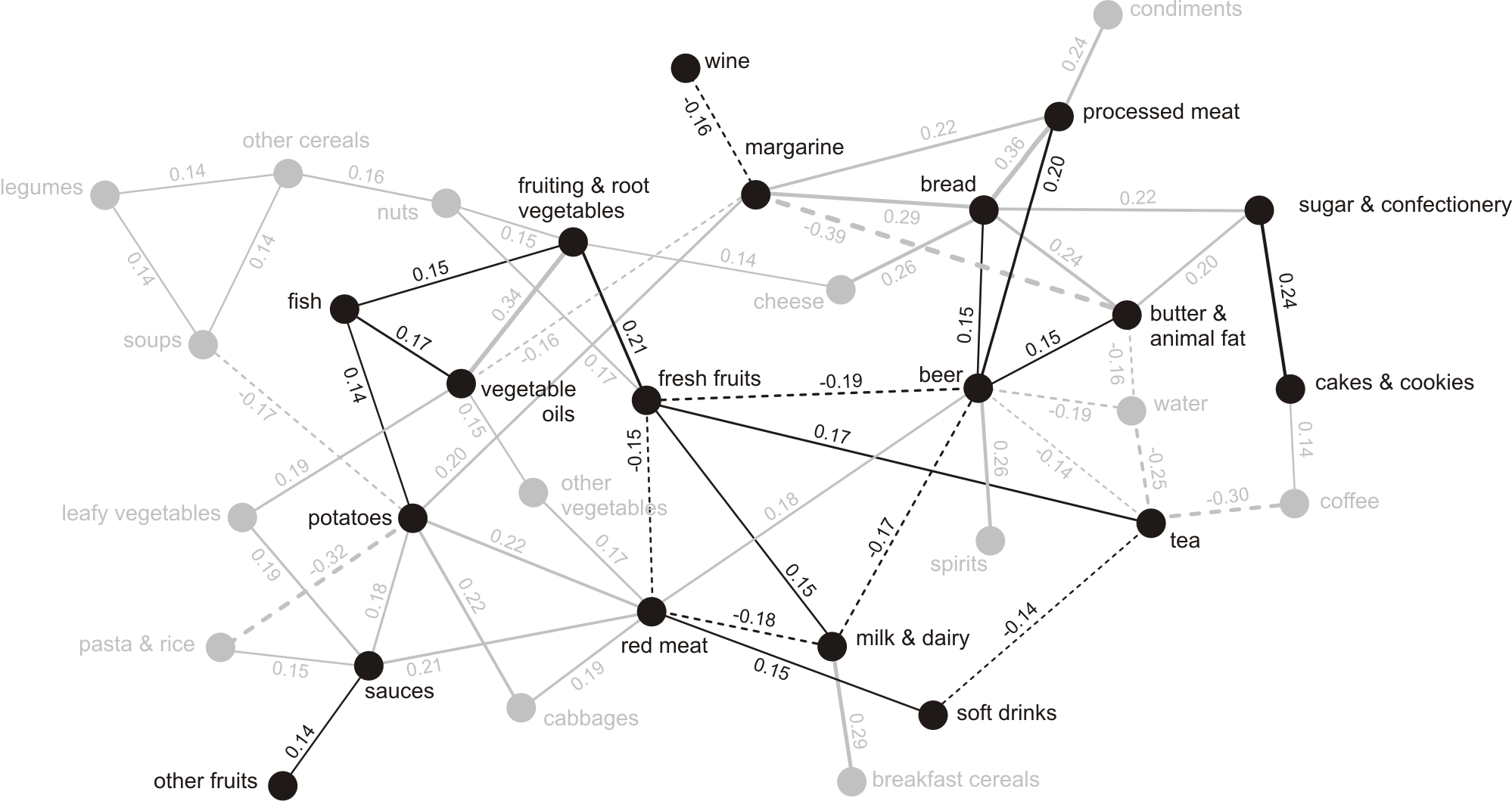


S3 Fig E: Habitual network emphasizing relations not found in any of the meal-specific dietary networks. 31% of relations were not present in any of the meal-specific dietary networks. Edges represent conditional dependencies between food groups revealed by partial correlation coefficients. The absence of an edge between 2 food groups indicates conditional independence between them. Continuous edges show positive partial correlations while broken edges show negative partial correlations. Line thickness is proportional to the strength of the correlations between food groups.
